# A homozygous splice-site variant in SAMHD1 shows variable expressivity of Aicardi-Goutières syndrome type 5: a case report and literature review

**DOI:** 10.3389/fped.2026.1787581

**Published:** 2026-05-15

**Authors:** Hammad Yousaf, Zehra Zonash, Javeria Manzoor, Asmat Ali, Lubaba Bintee Khalid, Ghazala Zafar, Sajid Ali, Mathias Toft, Ambrin Fatima, Zafar Iqbal

**Affiliations:** 1 Department of Biological and Biomedical Sciences, The Aga Khan University, Karachi, Pakistan; 2 Aga Khan Medical College, Karachi, Pakistan; 3 Institute of Clinical Medicine, University of Oslo, Oslo, Norway; 4 Department of Neurology, Oslo University Hospital, Oslo, Norway; 5 Centre for Regenerative Medicine and Stem Cells Research, The Aga Khan University, Karachi, Pakistan

**Keywords:** AGS5, interferonopathy, SAMHD1, variable expressivity, whole-exome sequencing

## Abstract

**Background:**

Aicardi-Goutières syndrome type 5 (AGS5) is a rare pediatric-onset monogenic interferonopathy caused by loss-of-function variants in the *SAMHD1* gene. Affected individuals typically present in infancy with microcephaly, leukodystrophy, intracranial calcifications, developmental delay, and spasticity. However, phenotypic heterogeneity and atypical systemic manifestations have been increasingly recognized.

**Case presentation:**

We report a consanguineous Pakistani family with two siblings affected by AGS5. The affected individuals (9-year-old female and 7-year-old male) presented early-infantile-onset neurodevelopmental delay, microcephaly, dysarthria, bilateral lower-limb muscular atrophy, and mild planovalgus deformity. They never attained independent ambulation and had only rudimentary speech. Notably, neuroimaging findings, spasticity, seizures, and aggressive behavior were absent. Whole-exome sequencing identified a homozygous canonical splice donor site variant in *SAMHD1* (NM_015474): c.1503 + 1G > A, predicted to abolish normal splicing and classified as pathogenic by the ACMG criteria (i.e., PVS1, PM2, PP1). Segregation analysis confirmed an autosomal recessive mode of inheritance, with both parents being heterozygous carriers and affected individuals homozygous for the variant.

**Conclusion:**

This report expands the mutational and phenotypic spectrum of *SAMHD1*-related AGS. The identification of a homozygous canonical splice donor site variant in a consanguineous South Asian family underscores the importance of considering AGS in children with congenital microcephaly and progressive neurodevelopmental impairment, even in the absence of intracerebral findings, chilblains, and spasticity.

## Introduction

Aicardi-Goutières syndrome (AGS) is a genetically heterogeneous, type I interferon-mediated leukodystrophy that typically presents in infancy with microcephaly, developmental delay, spasticity, leukodystrophy, and intracranial calcifications, often mimicking a congenital viral infection (1, 2). Genetically, AGS is classified into nine subtypes based on the underlying causative gene. These include AGS1, resulting from pathogenic variants in *TREX1*; AGS2, caused by *RNASEH2B* defects; AGS3, linked to *RNASEH2C*; AGS4, to *RNASEH2A*; AGS5, to *SAMHD1*; AGS6, to *ADAR1*; and AGS7, associated with deleterious variants in *IFIH1*; AGS8, to *LSM11*; and AGS9, to *RNU7-1* (1, 3). Regardless of the molecular cause, these subtypes converge on the defective pathways that control nucleic-acid metabolism and innate immune sensing, although phenotypic variability occurs between genotypes and within the same genotype (4, 5).

Of these subtypes, Sterile Alpha Motif and Histidine Aspartate Domain-containing Protein 1 (*SAMHD1*), causing AGS5, is a key regulator of intracellular deoxynucleoside triphosphate (dNTP) homeostasis, functioning as a triphosphohydrolase that degrades excess dNTPs to maintain the balanced pool required for accurate DNA replication and genome stability (6, 7). Because dNTP availability directly influences DNA synthesis and repair, tight control of these substrates is crucial for normal cellular function (8). Dysfunction of SAMHD1, through its central role in maintaining dNTP homeostasis, leads to secondary accumulation of endogenous nucleic acids within the cells. These aberrant nucleic acids in turn activate innate immune sensing pathways, including cGAS-STING pathway, driving a chronic, interferon-mediated inflammatory response. This pathogenic cascade underlies the clinical phenotype caused by *SAMHD1* mutations, which often mimics congenital viral infection and exhibits variable expressivity driven by underlying molecular heterogeneity (9–11).

Similar to several other immune-regulatory genes (12, 13), pathogenic variants in *SAMHD1* have been associated with recessive disease, while dominant disease phenotypes have also been reported, though this remains provisional. The clinical outcome of *SAMHD1* disruption depends on the underlying molecular mechanism: biallelic pathogenic variants lead to Aicardi-Goutières syndrome type 5 (AGS5, MIM#612952), while heterozygous variants are provisionally linked to chilblain lupus 2 (CHBL2, MIM#614415). These mechanistically distinct *SAMHD1*-associated disorders encompass a clinical spectrum ranging from AGS5 which is classically characterized by neuroimaging triad of intracranial calcifications (particularly in the basal ganglia and periventricular regions), leukodystrophy, and cerebral atrophy, along with microcephaly, delayed development, truncal hypotonia, spasticity, and irritability as reported in literature (14, 15) to predominantly autoimmune manifestations such as isolated chilblain lesions (16, 17). Increasing recognition of milder phenotypes further expands the known clinical spectrum of *SAMHD1*-associated disease (1, 7, 18). In a large AGS cohort, Rice et al. identified 27 distinct pathogenic *SAMHD1* variants, emphasizing significant allelic heterogeneity within this condition (19). Collectively, the expanding clinical and genetic spectrum of *SAMHD1*-associated disorders highlights the importance of careful genotype-phenotype correlation and recognition of both classical and non-classical presentations.

Here we report a consanguineous Pakistani family with two affected individuals harboring a biallelic splice donor site variant in *SAMHD1* (NM_015474.4): c.1503 + 1G > A, p.(?), underlying global developmental delay, muscular atrophy, mild intellectual disability, and unremarkable brain magnetic resonance imaging (MRI). Through a comprehensive literature review, we further explore gaps in understanding genotype-phenotype correlations, variability in neuroimaging findings, and diagnostic challenges associated with *SAMHD1*-related disease, particularly highlighting the absence of classical neuroimaging features as evidence of expanding phenotypic variability.

## Case presentation

The proband (IV:4) was a 9-year-old female, the Fourth-born child of consanguineous parents of Pakistani origin. Pregnancy and delivery were uneventful, and she was born at term with no perinatal complications. Neurological problems were evident from early infancy, marked by poor feeding (*HP:0011968*) and irritability (*HP:0000737*), followed by delayed developmental milestones (*HP:0001263*): unsupported sitting was achieved at 18 months, independent ambulation was never attained (*HP:0002540*), and by 1.5–2 years of age her speech was limited to simple babbling, and worsened over time (*HP:0000750*), and mild intellectual disability *(HP:0001256)*. At the most recent evaluation, head circumference measured 46 cm, consistent with microcephaly (*HP:0000252*). Brain MRI was unremarkable with no indications of leukodystrophy (*HP:0002415*) or cerebral atrophy (*HP:0002059*), while intracranial calcifications *(HP:0002514)* cannot be excluded since brain CT imaging was not available. Neurological examination revealed dysarthric speech (*HP:0001260*), bilateral lower-limb muscular atrophy (*HP:0003202*) with normal plantar reflexes, and mild bilateral planovalgus deformity (*HP:0001763*). Notably, there was no history of seizures (*HP:0001250*), spasticity *(HP:0001257)*, or behavioral dysregulation (*HP:0000708*).

Family history was significant for two similarly affected siblings. Individual IV:3 passed away in early childhood, was reportedly developmentally delayed, and no biological specimen was available for testing. The younger brother (IV:5) is alive with overlapping clinical manifestation comprising of delayed achievement of developmental milestones, microcephaly dysarthria, and bilateral mild planovalgus deformity (Table 1). Four siblings remain clinically unaffected.

**Table 1:** Summary of the individuals with *SAMHD1* recessive variants.

Family (Ref)	Individual	Zygosity	Variant	Microcephaly	Skin Findings (C/D/S)	Delayed Development	Spasticity	Brain Findings (ICC/DWMH/L)	Seizures	Additional Features	Additional Diagnoses
Current	31	Hom	c.1503 + 1G > A, p.(?)	Y	NA/NA/NA	Y	N	NA/NA/NA	N	Mild ID, LL muscular atrophy, Arthropathy	
Current	30	Hom	c.1503 + 1G > A, p.(?)	Y	NA/NA/NA	Y	N	NA/NA/NA	N	Mild ID, LL muscular atrophy, Arthropathy	
20 (38)	29	Hom	c.868 C > T, p.(Arg290Cys)	Y	NA/NA/NA	NA	NA	Y/NA/NA	NA	Congenital glaucoma, Congenital hypothyroidism, Strabismus, Delayed myelination, Truncal Hypotonia, Peripheral Hypertonia, Irritability	Moyamoya syndrome
19 (30)	28	Hom	Exon 1 del (g.36948791_36957774del)	NA	Y/NA/NA	NA	NA	Y/NA/NA	NA	Neurological impairments, Basal ganglia lacunar infarcts, Panniculitis, Lipoatrophy, Myositis, Contractures	
18 (28)	27	Hom	c.1324C > T, p.(Arg442*)	Y	Y/N/N	N	NA	Y/NA/NA	Y (T)		Idiopathic hypoparathyroidism
17 (37)	26	Hom	c.66delC, (p.Ser23Glnfs*43)	Y	Y/NA/NA	NA	Y	NA/NA/Y	NA	Hypertrichosis, Synophrys, Arched palate, Strabismus, Cryptorchidism, Cerebral + Cerebellar microinfarcts, Thrombocytopenia	Cornelia de Lange Syndrome, Scoliosis, Ischemic colitis
16 (33)	25	CH	c.676C > T, p.(Arg226Cys); c.1608 + 2T > C, p.(?)	N	N/NA/NA	N	NA	NA/NA/NA	NA	Unilateral exophoria, Bilateral cerebral microbleeds	Moyamoya disease, Mitral Valve chorda tendinae rupture (previously)
15 (27)	24	Hom	c.434G > C, p.(Arg145Pro)	NA	NA/NA/NA	Y	Y	Y/NA/NA	Y (G)	Ventricular dilatation, Leukodystrophy, Hypoplasia of corpus callosum	Triple X Syndrome, Infantile Cerebral Palsy, Hip dysplasia, Juvenile dermatomyositis
14 (24)	23	Hom	Exact variant not mentioned	Y	NA/NA/NA	Y	Y	Y/NA/NA	Y (T)	Dysmorphic facial features, LVH & Ascending aorta dilatation, Apnea + Snoring + Sweating in sleep, Cystic encephalomalacia, Gracile + Narrowed ACA & MCA, Nystagmus, Poor Feeding, Irritability, Thrombocytopenia	
13 (25)	22	CH	c.494T > C, p.(Phe165Ser); c.703C > T, p.(Gln235*)	Y	N/NA/NA	Y	NA	Y/Y/Y	NA	Pyramidal and extrapyramidal symptoms, Nystagmus, Poor Feeding, Truncal Hypotonia, Peripheral Hypertonia, Irritability	
12 (26)	21	Hom	c.1411–2A > G, p.(?)	Y	NA/NA/NA	NA	NA	NA/Y/NA	Y (TC)	Diffuse hypertonia, Irritability, Thrombocytopenia	Williams Syndrome, Moyamoya syndrome
11 (35)	20	CH	c.1270G > C, p.(Asp424His); c.658C > T, p.(Arg220*)	Y	NA/NA/NA	Y	NA	Y/NA/NA	NA	Frontal lobe lesions and cyst, Poor Feeding, Contractures, Thrombocytopenia	Generalised vitiligo, Alopecia areata, Encephalopathy, Cerebral palsy, Immune Thrombocytopenia, Autoimmune thyroid disease
10 (31)	19	Hom	c.490C > T, p.(Arg164*)	NA	Y/NA/Y	NA	NA	Y/NA/NA	NA	Subclinical hypothyroidism, Mild ID	
9 (15)	18	CH	c.602T > A, p.(Ile201Asn); c.1293A > T, p.(Leu431Phe)	NA	Y/NA/NA	Y	Y	Y/NA/NA	Y	Strabismus, Persistent erythema on toes, WM hyperintensity, Leukodystrophy, Truncal Hypotonia, Peripheral Hypertonia	
8 (36)	17	Hom	c.1265T > A, p.(Leu422Gln)	Y	NA/NA/NA	Y	Y	NA/NA/NA	Y (M)	Opisthotonus, Glaucoma, Hypertension, Recurrent fevers, Hepatosplenomegaly, Subcortical cysts, Frontal encephalocele, Cavum septum pellucidum, Thin corpus callosum, Cortical + Brainstem + Cerebellar atrophy, Poor Feeding, Contractures	
7 (32)	16	Hom	Del (Exact coordinates not reported)	Y	NA/NA/NA	NA	NA	Y/NA/NA	NA	Poor Feeding, Peripheral Hypertonia	
7 (32)	15	Hom	Del (Exact coordinates not reported)	NA	NA/NA/NA	Y	NA	NA/NA/NA	NA		
6 (22)	14	Hom	9 kb Del spanning Promoter, Exon 1, and Intron 1*	Y	NA/NA/NA	Y	NA	Y/NA/NA	Y (F,G)	Lateral Ventricle Dilatation, Subependymal cysts, Leukomalacia, Subacute cerebral infarcts, Intracerebral vasculopathy, Poor Feeding, Truncal Hypotonia, Peripheral Hypertonia, Irritability	
6 (22)	13	Hom	9 kb Del spanning Promoter, Exon 1, and Intron 1*	Y	NA/NA/NA	Y	NA	NA/NA/NA	NA	Delayed myelination, Hypoplasia of corpus callosum, Severe cortical atrophy, Global brain destruction, Poor Feeding, Irritability	
6 (22)	12	CH	c.1106T > C, p.(Leu369Ser); 9kb Del spanning Promoter, Exon 1, and Intron 1*	Y	NA/NA/NA	Y	Y	Y/NA/NA	NA	Bilateral calcified thalamic vessels	
6 (22)	11	CH	c.649insG, p.(Phe217CysfsTer2); 9 kb Del spanning Promoter, Exon 1, and Intron 1*	Y	NA/NA/NA	Y	NA	NA/NA/NA	NA	History of right ICA thrombosis	
5 (23)	10	Hom	c.626–1G > C, p.(?)	N	N/NA/NA	Y	Y	Y/NA/NA	NA	Cerebral atrophy, Small testes, Vertebral osteoporosis, Axonal neuropathy, Myopathy	Heterozygous WRN mutation
4 (34)	9	CH	c.869G > A, p.(Arg290His); c.1642C > T, p.(Gln548*)	Y	Y/Y/Y	Y	Y	Y/NA/NA	Y (M,T)	Muscular hypotonia, Hypersonia,Hepatomegaly, Opisthotonus, Extrapyramidal dyskinesia, Frontal cerbral atrophy, WM cystic degeneration, Mouth Ulcers, Poor Feeding, Contractures, Arthropathy, Irritability, Thrombocytopenia	SLE, Scoliosis (severe)
3 (16)	8	Hom	c.602T > A, p.(Ile201Asn)	N	Y/NA/NA	Y	Y	Y/NA/NA	NA		Moyamoya syndrome
3 (16)	7	Hom	IVS13 + 1G > T, p.(?)	Y	Y/NA/NA	Y	NA	Y/NA/NA	Y (G)	Raised IOP, Extensive right MCA + ACA infarcts, Contractures, Peripheral Hypertonia	Cerebral palsy
3 (16)	6	CH	c.427C > T, p.(Arg143Cys); c.602T > A, p.(Ile201Asn)	Y	Y/NA/NA	Y	NA	Y/NA/NA	NA	Ventriculomegaly, Intracerebral Anuerysms	Leukocytoclastic vasculitis
3 (16)	5	Hom	Del Exons 12–16	NA	NA/NA/NA	NA	Y	Y/Y/Y	Y (G)	GER, Strabismus, Ventricular enlargement, Intracerebral anuerysms, Poor Feeding, Truncal Hypotonia, Peripheral Hypertonia	Leukocytoclastic vasculitis
3 (16)	4	CH	c.649insG, p.(Phe217CysfsTer2); 9 kb Del spanning Promoter, Exon 1, and Intron 1	Y	Y/Y/Y	Y	NA	Y/NA/NA	NA	Brain atrophy, Severe ID, Tetraplegic, Raynaud Phenomenon, Cutis marmorata, Lacunar infarcts of Basal Ganglia, Bilateral ICA stenosis, Obstruction of basilar artery + right vertebral artery, Narrowing of left MCA, Poor feeding	Moyamoya syndrome (suspected)
2 (29)	3	Hom	Del Exons 1–13	Y	Y/NA/Y	Y	Y	Y/NA/NA	NA	Unilateral open angle glaucoma, Recurrent fevers, Facial erythema, Peripheral sensory neuropathy, Hepatosplenomegaly, Peripheral Hypertonia	
1 (21)	2	CH	c.433C > T, p.(Arg145*); c.490C > T, p.(Arg164*)	Y	Y/Y/Y	Y	NA	N/N/NA	NA	Osteopenia, Mouth Ulcers, Arthropathy	
1 (21)	1	CH	c.433C > T, p.(Arg145*) c.490C > T, p.(Arg164*)	N	Y/Y/Y	N	Y(LL)	NA/NA/NA	NA	Mouth Ulcers	

C, chilblains (*HP:0009710*); D, dry skin (*HP:0000958*); S, scaly skin (*HP:0040189*); ICC, intracerebral calcifications (*HP:0002514*); DWMH, deep white matter hypodensities (*HP:0007321*); L, leukoencephalopathy (*HP:0002352*); LL, lower limbs; T, tonic; TC, tonic-clonic; M, myoclonic; F, focal; G, generalized; Del, deletion; ID, intellectual disability; Hom, homozygous; CH, compound heterozygous.

* this deletion also impacts 3’ end of *RBL1.*

**Figure 1:**
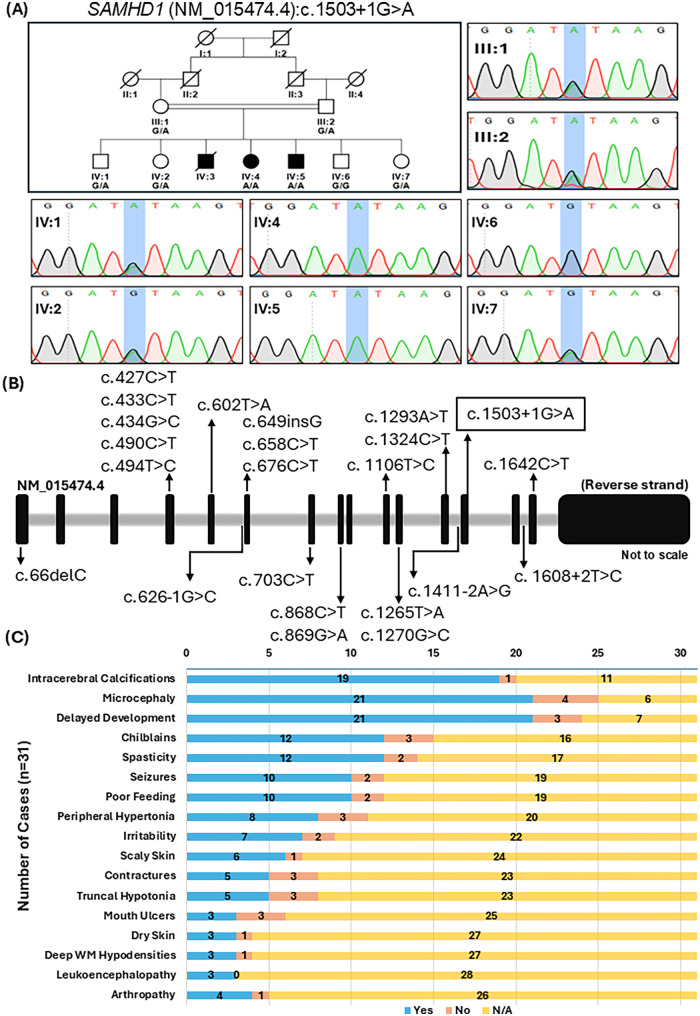
Biallelic splice-site variant causes AGS5 in a Pakistani kindred. **(A)**: Top: Pedigree of the investigated family presenting symptoms of Aicardi-Goutières Syndrome 5; filled boxes represent affected individuals, double lines indicate consanguinity. *SAMHD1*: c.1503 + 1G > A genotype indicated below individuals: G/G (wild-type), G/A (heterozygous), A/A (homozygous). Representative chromatograms are given on the right and at the bottom. **(B)**: Schematic representation of the gene structure of *SAMHD1.* A flipped diagram of the coding reverse strand, consisting of 16 exons (represented by solid filled boxes) and 15 introns (represented by grey filled area between the boxes). Homozygous and compound heterozygous variants resulting in the AGS5 phenotype available in literature (Table 1) have been marked along the structure. Large deletions are not marked. The proband's homozygous splice-site variant (c.1503 + 1 G > A) is indicated by a black box. The mutation occurs at a canonical donor splice-site of exon 13. Another mutation (c.1503 + 1 G > T) has also been reported at this site previously (4). **(C)**: A schematic showing commonly reported symptoms by reports of AGS5 attributed to *SAMHD1* homozygous deleterious variants. N/A was recorded where it was not explicitly stated whether a patient had a particular symptom.

Whole-exome sequencing (WES) of the proband was performed as described earlier (12) which identified a homozygous canonical splice donor site variant in *SAMHD1* (NM_015474.4): c.1503 + 1G > A, p.(?). The variant abolishes the consensus splice donor site and is predicted to result in aberrant splicing and likely loss-of-function. According to ACMG guidelines (20), it fulfills PVS1 (canonical splice-site loss in a gene with established loss-of-function mechanism), PM2 (absent from control populations), and PP1 (supporting; segregation in affected family members) and is therefore classified as pathogenic. The overall population frequency of this variant is 0.0.00000313 in gnomAD (v4.1.0) and is absent from South Asian population (gnomAD v4.1.0). Segregation analysis confirmed both parents and healthy siblings as heterozygous carriers and the affected individuals as homozygous for the variant (Figure 1A).

## Discussion

Biallelic pathogenic variants in *SAMHD1* are known to cause AGS5, and the clinical and molecular spectrum has been characterized as (19) typically presenting with characteristic neuroimaging findings, neurodevelopmental impairments, microcephaly, spasticity, seizures, and skin manifestations along with some less common features (Figures 1C and Table 1). The current study describes a consanguineous Pakistani family with two siblings harboring a homozygous canonical splice donor site variant in *SAMHD1* (c.1503 + 1G > A) which is predicted to disrupt normal splicing and likely result in loss of function. The affected individuals presented progressive early-infantile-onset global developmental impairment, microcephaly, muscular atrophy, poor feeding, and irritability while features such as neuroimaging findings, spasticity and seizures were absent. However, the partially overlapping clinical features with previously reported cases, the known variable expressivity associated with biallelic *SAMHD1* variants (21, 22), segregation of the variant within family, and extremely low frequency in gnomAD together support this variant as the most likely molecular cause of the disease in these patients. These findings therefore highlight a non-classical presentation of AGS5 and demonstrate variable expressivity associated with biallelic *SAMHD1* variants.

The affected individuals in the current study harbor a consensus splice-site variant in *SAMHD1*: c.1503 + 1G > A. A pathogenic homozygous G > T variant at c.1503 + 1 site has previously been reported in a patient with neurological symptoms, although the age of onset and detailed clinical features was not specified (4). Unlike the cases investigated in our study, individuals with *SAMHD1* variants in the said study displayed classical clinical presentation, including intracranial calcifications, increased intracranial pressure (*HP:0002516*), chilblains (*HP:0009710*), generalized hypertonia (*HP:0001276*), glaucoma (*HP:0000501*), seizures, and vascular involvement. However, the lack of detailed clinical information for the individual carrying the variant at the exact site precludes direct comparison with our cases. Among recessive *SAMHD1* mutations reported so far, missense variants predominate (*n* = 11), followed by stop-gained variants (*n* = 6), with splice-site variants and deletions occurring at similar frequencies (*n* = 5 each), and frameshift variants being comparatively rare (*n* = 2). Reported *SAMHD1* variants occur across multiple regions of the gene, with no clear mutational hotspot identified to date (Figure 1B). The SAMHD1 protein comprises three functional domains: an N-terminal SAM domain mediating protein interactions, a catalytic HD domain essential for dNTPase activity, and a C-terminal domain involved in protein stability and regulation (7). Given the presence of three domains with distinct functional roles and the diversity of reported variant types, the observed phenotypic variability may reflect differences in mutation type, domain involvement, and the degree of functional disruption. However, due to the relatively small number of cases described to date, genotype-phenotype correlations in AGS5 remain incompletely understood.

## Review of literature

A narrative literature review of case reports and series was conducted using the PubMed database. The search string “SAMHD1” AND (“Aicardi-Goutières syndrome type 5” OR “AGS5” OR “Aicardi-Goutières syndrome” OR “AGS”) was applied, with filters for articles published between 2010 and 2025, human subjects, and English language. Reports only describing AGS types other than AGS5 were excluded, and only studies reporting recessive variants were included.

The search yielded 29 cases of AGS5 harboring homozygous or compound heterozygous *SAMHD1* variants (Table 1) (14, 15, 21–38). The current study increases the total number of reported cases to 31. To date, cases have been nearly equally distributed between males and females, with 18/31 (58%) males and 13/31 (42%) females. The most common symptoms reported included intracerebral calcifications, microcephaly, and delayed development (Figure 1C). These are largely consistent with the classical neurological findings of AGS5 in early infancy, which reportedly also include leukodystrophy, spasticity, and cerebral atrophy (1, 2). However, collated data from these studies found a relatively low incidence of these features with spasticity in 12/31 (39%), neural structural atrophy in 6/31 (19%), and leukodystrophy in 3/31 (10%) individuals (Table 1). Such variability may arise from different mutation types and consequent residual protein function, differences in genetic background, or the influence of additional modifying factors. Further functional and clinical studies will be required to better understand the mechanisms underlying this phenotypic variability.

Interestingly, data also showed presentation of 4 cases with thyroid gland involvement (*HP:0000820*) and delayed myelination (*HP:0012448*) of neural structures each, 5 cases with cerebral infarcts (*HP:0025722*), and 3 cases with glaucoma (*HP:0000501*)—features not *typically* associated with AGS5. Furthermore, frequently reported co-morbid conditions included Moyamoya syndrome (4/31 cases), leukocytoclastic vasculitis and cerebral palsy (2/31 each), while Cornelia de Lange syndrome, Triple X syndrome, Williams syndrome, and juvenile dermatomyositis were each reported in a single case. Although vascular findings are not commonly associated with AGS5, review of reported cases noted 14 out of 31 individuals with vascular anomalies, including aortic dilation (22), narrowed anterior and/or middle cerebral arteries (15, 22), cerebral microbleeds or infarcts (15, 22, 28, 30, 37), calcified thalamic vessels (22), internal carotid artery thrombosis (22), internal carotid artery stenosis (15), obstruction of basilar and vertebral arteries (15), intracerebral aneurysms (15). Of these, one had an overlapping diagnosis of Moyamoya Syndrome (33), another had a suspected diagnosis of Moyamoya Syndrome (15), and 2 had leukocytoclastic vasculitis (15) (Table 1). In cases with isolated *SAMHD1* mutations and no other likely explanation for the co-existing diagnoses, the observed propensity of vascular anomalies suggests a potential role of *SAMHD1* in the structural integrity of the vascular system. However, this remains speculative, as no direct evidence currently links *SAMHD1* to vascular development or function, and the co-occurrence of these anomalies could be entirely coincidental.

## Conclusion

Our report highlights several important considerations. First, the identification of a novel splice-site variant emphasizes the utility of genomic sequencing in consanguineous populations, where rare recessive alleles may be enriched. Second, the absence of frequently reported features associated with AGS5 such as intracranial findings, seizures, and spasticity in the patients underscores that AGS5 should be considered even in children with isolated congenital microcephaly and neurodevelopmental delay without full phenotypic presentation. Finally, based on the literature review findings of concomitant diagnoses pertaining to systems other than neurological, perhaps a lower threshold for suspicion should be kept for considering a diagnosis of AGS5, given that the “classical” presentation is often not present.

## Data Availability

The original contributions presented in the study are included in the article/Supplementary Material, further inquiries can be directed to the corresponding authors.
